# Temperament Clusters in a Normal Population: Implications for Health and Disease

**DOI:** 10.1371/journal.pone.0033088

**Published:** 2012-07-18

**Authors:** Jaana Wessman, Stefan Schönauer, Jouko Miettunen, Hannu Turunen, Pekka Parviainen, Jouni K. Seppänen, Eliza Congdon, Susan Service, Markku Koiranen, Jesper Ekelund, Jaana Laitinen, Anja Taanila, Tuija Tammelin, Mirka Hintsanen, Laura Pulkki-Råback, Liisa Keltikangas-Järvinen, Jorma Viikari, Olli T. Raitakari, Matti Joukamaa, Marjo-Riitta Järvelin, Nelson Freimer, Leena Peltonen, Juha Veijola, Heikki Mannila, Tiina Paunio

**Affiliations:** 1 Helsinki Institute for Information Technology and Department of Computer Science, University of Helsinki, Helsinki, Finland; 2 Institute for Molecular Medicine Finland and National Institute for Health and Welfare, Helsinki, Finland; 3 Helsinki Institute for Information Technology and Department of Information and Computer Science, Aalto University, Espoo, Finland; 4 University of California Los Angeles Center for Neurobehavioral Genetics, University of California Los Angeles, Los Angeles, California, United States of America; 5 Institute of Health Sciences, University of Oulu, Oulu, Finland; 6 Department of Psychiatry, Helsinki University Central Hospital, Helsinki, Finland; 7 Finnish Institute of Occupational Health, Helsinki, Finland; 8 Unit of General Practice, University Hospital of Oulu, Oulu, Finland; 9 LIKES Research Center for Sport and Health Sciences, Jyväskylä, Finland; 10 Department of Psychology, University of Helsinki, Helsinki, Finland; 11 Department of Medicine, Turku University Central Hospital and University of Turku, Turku, Finland; 12 Department of Clinical Physiology, Turku University Central Hospital, and Research Center of Applied and Preventive Cardiovascular Medicine, University of Turku, Turku, Finland; 13 Tampere School of Public Health, University of Tampere, and Psychiatric Department, Tampere University Hospital, Tampere, Finland; 14 Department of Epidemiology and Public Health, Imperial College, London, United Kingdom and Department of Public Health Science and General Practice, University of Oulu, Oulu, Finland; 15 Wellcome Trust Sanger Institute, Hinxton, Cambridge, United Kingdom; 16 Department of Medical Genetics, University of Helsinki, Helsinki, Finland; 17 Department of Psychiatry, University of Oulu, Oulu, Finland; Umeå University, Sweden

## Abstract

**Background:**

The object of this study was to identify temperament patterns in the Finnish population, and to determine the relationship between these profiles and life habits, socioeconomic status, and health.

**Methods/Principal Findings:**

A cluster analysis of the Temperament and Character Inventory subscales was performed on 3,761 individuals from the Northern Finland Birth Cohort 1966 and replicated on 2,097 individuals from the Cardiovascular Risk in Young Finns study. Clusters were formed using the k-means method and their relationship with 115 variables from the areas of life habits, socioeconomic status and health was examined.

**Results:**

Four clusters were identified for both genders. Individuals from Cluster I are characterized by high persistence, low extravagance and disorderliness. They have healthy life habits, and lowest scores in most of the measures for psychiatric disorders. Cluster II individuals are characterized by low harm avoidance and high novelty seeking. They report the best physical capacity and highest level of income, but also high rate of divorce, smoking, and alcohol consumption. Individuals from Cluster III are not characterized by any extreme characteristic. Individuals from Cluster IV are characterized by high levels of harm avoidance, low levels of exploratory excitability and attachment, and score the lowest in most measures of health and well-being.

**Conclusions:**

This study shows that the temperament subscales do not distribute randomly but have an endogenous structure, and that these patterns have strong associations to health, life events, and well-being.

## Introduction

Temperament refers to early-appearing individual differences in emotional responding and central to its definition is the notion that temperament is innate [1]. There is now substantial data supporting the biologically based nature of these individual differences in emotional experience and regulation, with heritability estimates ranging from 50 to 65% [2], [3], [4], [5]. These stable and organized patterns of behavioral responses across a range of contexts are thus believed to form the basis of more complex psychological structures.

**Figure 1:**
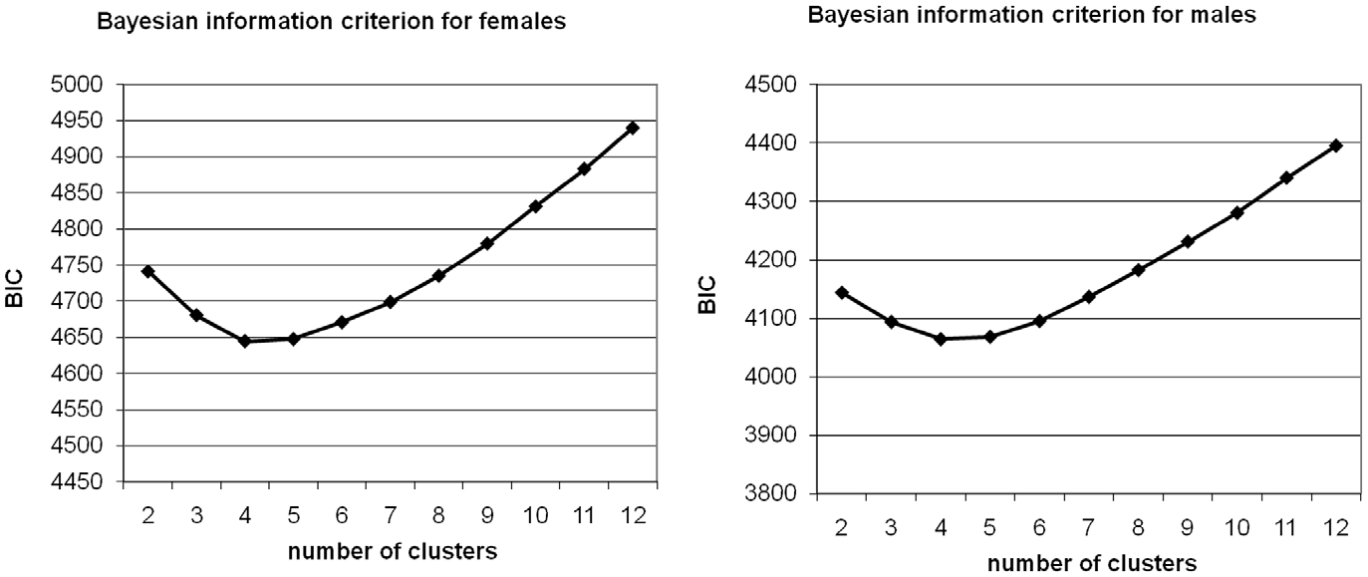
Bayesian information criterion (BIC) scores for various cluster sizes. Clustering of the subscales showed stable results with an optimum of four clusters in both females (left) and males (right).

Several models have been proposed for classifying temperament, including Cloninger’s psychobiological model of four higher-order temperament dimensions distinguished by their stimulus-response characteristics [6], [7]. The widely used Temperament and Character Inventory (TCI) [7] measures individual differences along four main temperament dimensions: novelty seeking (NS), harm avoidance (HA), reward dependence (RD) and persistence (P). NS is a tendency to respond with intense excitement to novel stimuli, or cues for potential rewards or potential relief of punishment and thereby activating behavior. HA is a tendency to respond intensively to signals of aversive stimuli, thereby inhibiting behavior. RD is a tendency to respond intensely to signals of reward, especially social rewards, thereby maintaining and continuing particular behaviors. P is a tendency to persevere in behaviors that have been associated with reward or relief from punishment. Scores measured by the TCI distribute normally in the population with sex-dependent differences [8].

**Figure 2:**
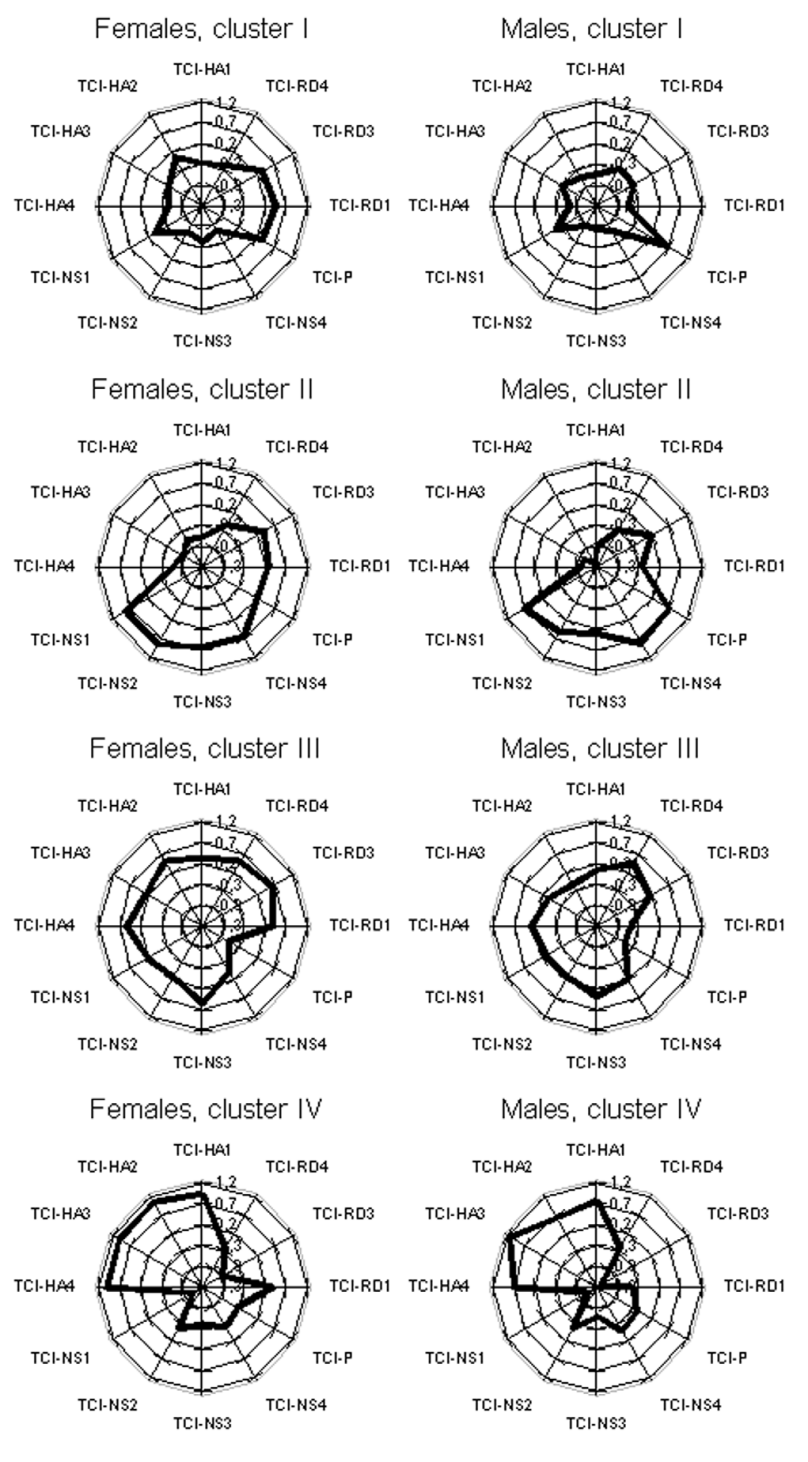
Star plots describing the clusters. Cluster results for females (left column) and males (right column) are presented as star plots, with 0 as the sample mean and 1 as the sample standard deviation. The average score of each cluster (I-IV) on each of the twelve TCI subscales are indicated by the thick black line, with the line closer to the middle of the plot representing lower scores and the line closer to the edge of the plot representing higher scores.

The importance of temperament to mental health [7] and to some extent to somatic health [9], [10] has been previously established. In particular, high levels of HA are associated with a number of psychiatric disorders [11], [12], [13], [14], [15]. A more complete understanding of the influence of temperament on health outcome will require dissection of the specific pathways between temperament and outcome. However, despite the original hypothesis that temperament dimensions are independent behavioral systems [6], accumulating evidence suggests correlations between dimensions [16]. Organizing personality traits as profiles or clusters (person-oriented approach) provides an opportunity to examine the context of an individual’s traits, as opposed to considering individual traits in isolation (variable-centered approach), and does not require the assumption that personality dimensions operate independently (Crockett et al., 2006). While there is growing support that such personality clusters predict a number of health outcomes, much of this work has been focused on clusters defined in childhood or adolescence (e.g., Crockett et al., 2006) or has been focused on a single diagnostic outcome (e.g., Muthen & Muthen, 2000).

**Table 1:** Number and proportion of variables significantly associated with clusters, any scale, and each individual scale, after correction for multiple testing.

	Females		Males	
Grouping	Number	Proportion	Number	Proportion
Clusters	47	0.41	42	0.37
Any Scale	48	0.42	51	0.44
HA1	24	0.21	29	0.25
HA2	24	0.21	21	0.18
HA3	27	0.23	30	0.26
HA4	31	0.27	30	0.26
NS1	11	0.096	19	0.17
NS2	6	0.052	4	0.035
NS3	13	0.11	15	0.13
NS4	8	0.07	4	0.035
P	4	0.035	5	0.043
RD1	11	0.096	14	0.12
RD3	20	0.17	19	0.17
RD4	9	0.078	6	0.052

In order to examine the inter-relationships between dimensions of temperament, as defined by Cloninger’s model, and relationships between temperament and adult outcome, we conducted a series of analyses in the Northern Finland Birth Cohort 1966. This longitudinal birth-cohort provided the opportunity to assess the relationship between profiles of temperament and overt expression of psychiatric illness, health outcome, and lifestyle in a large, relatively genetically homogeneous population.

**Table 2:** Means and standard deviations from 15D questionnaire for females.

	Clusters								Subscales		
	I		II		III		IV		First^a^	Second^b^	Other^c^
	Mean	SD	Mean	SD	Mean	SD	Mean	SD			
Mobility	1.017	0.169	1.031	0.173	1.044	0.214	1.039	0.193			
Vision	1.081	0.342	1.083	0.330	1.070	0.262	1.109	0.381			
Hearing	1.029	0.179	1.056	0.255	1.055	0.266	1.063	0.262			
Breathing*	1.096	0.338	1.136	0.399	1.179	0.429	1.191	0.429			
Sleeping***	1.250	0.480	1.368	0.584	1.421	0.601	**1.433**	0.590	HA1	HA4	NS4
Eating	1.000	0.000	1.004	0.064	1.002	0.043	1.010	0.098			
Speech***	1.029	0.168	1.014	0.120	**1.031**	0.174	1.101	0.318			HA2 HA4 RD4
Elimination	1.160	0.406	1.186	0.439	1.203	0.425	1.196	0.438			
Usual activities*	1.027	0.194	1.043	0.214	1.060	0.246	1.089	0.294	HA4		
Mental function***	1.071	0.257	1.085	0.293	1.121	0.326	**1.196**	0.409	HA4		HA1 HA3
Discomfort and symptoms***	1.516	0.576	1.477	0.570	1.590	0.578	**1.661**	0.580	HA4	HA1	
Depression***	1.277	0.473	1.324	0.565	1.485	0.615	**1.652**	0.664			HA2 HA3 RD3
Distress***	1.215	0.421	1.254	0.506	1.375	0.555	**1.582**	0.665			HA2 NS1 RD1 RD3
Vitality***	1.329	0.513	1.357	0.588	1.497	0.601	**1.640**	0.652			HA3 RD3
Sexual activity**	1.104	0.353	1.081	0.288	1.161	0.424	**1.184**	0.472	HA4	HA1	

* *p*<0.05 uncorrected.

** *p*<0.05 corrected.

*** *p*<0.01 corrected.

a First scale: the individual TCI scale with the strongest significant association to the variable.

b Second scale: any other individual TCI scale with a significant association to the variable that is stronger than the clusters.

c Other scale(s): any other individual TCI scale that is significantly associated to the variable. For significant associations between clusters and variables from the 15D questionnaire, the mean value for the cluster with the highest scores are in bold.

Specifically, we hypothesized that individuals from this large birth cohort could be stratified into functionally meaningful groups according to their temperament profiles. As temperament is an important determinant for affective regulation and behavior, we hypothesized that individuals from these separate temperament groups would also differ in their life habits, socioeconomic status, and psychiatric and somatic health. To test these hypotheses, we performed a cluster-based analysis of responses on the TCI in 3,761 men and women from the Northern Finland Birth Cohort 1966, and tested for differences between the resulting clusters on a wide range of life domains. In order to test the stability of these clusters in a separate sample, we also replicated the results of the cluster structure analysis among 2,097 participants of the Cardiovascular Risk in Young Finns study.

**Table 3:** Means and standard deviations from 15D questionnaire for males.

	Clusters								Subscales			
	I		II		III		IV		First^a^	Second^b^		Other^c^
	Mean	SD	Mean	SD	Mean	SD	Mean	SD				
Mobility*	1.023	0.165	1.016	0.147	1.053	0.310	1.062	0.343				
Vision	1.094	0.357	1.060	0.279	1.056	0.229	1.091	0.324				
Hearing*	1.069	0.311	1.046	0.222	1.053	0.225	1.099	0.326				
Breathing	1.085	0.311	1.081	0.274	1.115	0.410	1.118	0.323	HA4			
Sleeping***	1.229	0.447	1.323	0.549	1.313	0.515	**1.444**	0.623			RD1	
Eating	1.005	0.068	1.000	0.000	1.000	0.000	1.019	0.186				
Speech***	1.046	0.241	1.019	0.137	1.016	0.127	**1.126**	0.349			NS1 RD3	
Elimination*	1.106	0.330	1.070	0.256	1.078	0.305	1.161	0.429	HA1			
Usual activities***	1.030	0.239	1.041	0.224	1.047	0.264	**1.126**	0.412	HA4	HA1		
Mental function***	1.067	0.285	1.049	0.216	1.082	0.275	**1.199**	0.433			HA1 HA3	
Discomfort and symptoms***	1.353	0.507	1.428	0.604	1.416	0.564	**1.553**	0.540	HA1			
Depression***	1.150	0.377	1.233	0.448	1.265	0.499	**1.617**	0.685			HA2 NS1 RD1 RD3	
Distress***	1.155	0.387	1.203	0.435	1.263	0.490	**1.566**	0.622			HA2 NS1 RD1 RD3	
Vitality***	1.180	0.430	1.233	0.454	1.307	0.524	**1.539**	0.662			HA2 HA3 NS1 RD1 RD3	
Sexual activity***	1.025	0.172	1.038	0.191	1.056	0.255	**1.143**	0.481			HA3 HA4	

* *p*<0.05 uncorrected.

** *p*<0.05 corrected.

*** *p*<0.01 corrected.

a First scale: the individual TCI scale with the strongest significant association to the variable.

b Second scale: any other individual TCI scale with a significant association to the variable that is stronger than the clusters.

c Other scale(s): any other individual TCI scale that is significantly associated to the variable. For significant associations between clusters and variables from the 15D questionnaire, the mean value for the cluster with the highest scores are in bold.

## Materials and Methods

### Participants and Measures

*The Northern Finland Birth Cohort 1966* (NFBC 1966) is a longitudinal one-year birth cohort originally including all males and females whose expected year of birth was in 1966 in Finland’s two northernmost provinces, Oulu and Lapland (N = 12,058 live-born individuals) [17]. The cohort members have been carefully monitored prospectively from the prenatal period onwards. The current study sample is based on cohort members who lived in Finland at the age of 16 years (N = 10,526∶5,365 male, 5,161 female), as validated psychiatric diagnoses from the Finnish Hospital Discharge Register are available for these subjects [18].

**Table 4:** Confirmed diagnoses.

	Clusters				Subscales
	I	II	III	IV	First^a^
**Females**					
Asthma	8.0%	11%	9.5%	7.1%	
Allergic rhinitis*	26%	29%	27%	**19**%	
Eczema*	38%	34%	38%	**30**%	
Allergic eye symptoms	20%	22%	21%	16%	
Hypertension	13%	14%	13%	15%	
Diabetes	2.2%	1.8%	1.8%	1.8%	
Thyroiditis	3.0%	3.0%	2.3%	1.8%	
Gastric ulcer	1.3%	1.4%	2.7%	2.1%	
Epilepsy	0.6%	2.2%	1.2%	1.4%	
Migraine	17%	21%	20%	20%	
Other neurologic disease	0.7%	0.2%	1.1%	1.6%	
Rheumatoid arthritis*	1.3%	0.6%	1.4%	**3.0**%	
Other arthritic condition	4.7%	5.6%	5.7%	4.4%	
Degenerative or other back condition	14%	15%	12%	13%	
Schizophrenia*	0.4%	1.2%	1.6%	**3.0%**	
*Depression* ***	2.4%	3.6%	3.7%	**8.5%**	HA4
**Males**					
Asthma*	7.7%	**13%**	7.2%	7.9%	
Allergic rhinitis	20%	19%	17%	22%	
Eczema	24%	24%	21%	23%	
Allergic eye symptoms	17%	14%	13%	14%	
Hypertension*	9%	12%	10%	**15%**	
Diabetes	0.2%	0.0%	0.8%	0.8%	
Thyroiditis	0.2%	1.3%	0.4%	0.8%	
Gastric ulcer	2.2%	2.6%	2.5%	3.4%	
Epilepsy	0.9%	0.8%	0.8%	2.6%	
Migraine	6.0%	6.0%	7.0%	7.9%	
Other neurologic disease	0.2%	0.3%	1.0%	1.1%	
Rheumatoid arthritis	0.7%	0.3%	0.8%	0.3%	
Other arthritic condition	4.7%	5.7%	5.8%	2.9%	
Degenerative or other back condition	17%	20%	19%	16%	
Schizophrenia**	0.9%	1.3%	2.0%	**3.7%**	
Depression***	1.1%	2.1%	2.1%	**5.5%**	HA4

* *p*<0.05 uncorrected.

** *p*<0.05 corrected.

*** *p*<0.01 corrected.

a First scale: the individual TCI scale with the strongest significant association to the diagnosis. For significant associations between clusters and diagnoses, the values for the cluster with the highest percentage are in bold.

In the 31-year follow-up of the cohort, all subjects alive at that time with a known address were sent a postal questionnaire (N = 10,526). For subjects then living in the Oulu or Lapland provinces or in Helsinki area (N = 8,463), the questionnaire included an invitation to take part in a clinical examination. The subjects who participated (N = 5,960 (70%): 2863 male (65%), 3097 female (76%)) were also asked to fill in another questionnaire on temperament, health, and occupation [19].

**Table 5:** Psychological scales for females.

	Clusters								Subscales		
	I		II		III		IV		First^a^	Second^b^	Other^c^
Females	Mean	SD	Mean	SD	Mean	SD	Mean	SD			
Schizoid scale***	2.5	1.2	2.5	1.2	2.8	1.4	**3.5**	1.3			NS1
Perceptional aberration scale***	2.1	2.8	2.4	2.9	2.3	3.1	**3.8**	4.4	HA1 RD1		HA2 HA3 HA4 NS2 NS4 P RD3 RD4
Physical anhedonia scale***	12.1	5.2	11.1	5.4	12.6	5.2	**16.0**	6.3			HA1 HA4 NS3 P RD1 RD4
Social anhedonia scale***	7.2	3.9	7.3	4.2	6.6	3.5	**12.5**	5.5			NS3
Bipolar scale***	10.4	4.5	11.7	4.8	10.7	4.8	**14.9**	5.1			
Hypomania personality scale***	11.0	6.3	**16.3**	7.5	9.6	6.3	10.1	6.0			HA1
Symptoms of anxiety***	1.3	0.2	1.3	0.3	1.3	0.3	**1.4**	0.4	HA1 HA4		HA2 HA3 NS2 RD1 RD3
Symptoms of depression***	1.3	0.3	1.3	0.4	1.4	0.4	**1.5**	0.4			HA2 HA3 RD1 RD3
Alexithymia, factor 1***	12.7	5.6	12.6	5.1	13.5	5.3	16.9	5.8			NS1 RD1 RD4
Alexithymia, factor 2***	12.1	3.6	11.7	3.1	11.9	3.1	14.6	3.1			HA1 HA4 NS3
Alexithymia, factor 3**	27.6	4.9	27.2	3.8	27.0	4.2	28.1	3.5	RD3		

* *p* < 0.05 uncorrected.

** *p*<0.05 corrected.

*** *p*<0.01 corrected.

a First scale: the individual TCI scale with the strongest significant association to the variable.

b Second scale: any other individual TCI scale with a significant association to the variable that is stronger than the clusters.

c Other scale(s): any other individual TCI scale that is significantly associated to the variable. For significant associations between clusters and variables from the psychological scales, the mean value for the cluster with the highest scores are in bold. Psychological scales included the Schizoid Scale (from the Minnesota Multiphasic Personality Inventory); *Perceptual Aberration Scale*, *Physical Anhedonia Scale* and *Social Anhedonia Scale; Bipolar II scale*; *Hypomanic Personality Scale*; *Symptoms of anxiety* and *symptoms of depression* from the HSCL-25; and Alexithymia factors 1–3 from the Twenty Item Toronto Alexithymia Scale (Factor 1: difficulties in identifying feelings; Factor 2: difficulties in describing feelings; and Factor 3: externally oriented thinking).

This study was limited to those individuals for whom a complete personality questionnaire had been returned and who had not been diagnosed with mental retardation (N = 3761 out of the 5084 who returned the questionnaire). Of all subjects who were provided the temperament questionnaire, 63% (60% of the males, 66% of the females) participated.

**Table 6:** Psychological scales for males.

	Clusters								Subscales		
	I		II		III		IV		First^a^	Second^b^	Other^c^
Females	Mean	SD	Mean	SD	Mean	SD	Mean	SD			
Schizoid scale***	1.9	1.2	2.1	1.3	2.2	1.4	3.0	1.5			NS1 NS2 RD3
Perceptional aberration scale***	1.3	2.1	2.3	3.4	1.6	2.5	3.0	3.9	RD1		HA1 HA4 NS2 NS4 RD3 RD4
Physical anhedonia scale***	18.0	6.7	15.0	6.4	17.5	6.6	21.9	7.9			HA1 HA2 HA4 NS3 P
Social anhedonia scale***	10.7	5.0	8.9	5.0	9.2	4.1	16.3	6.4			NS3 RD1
Bipolar scale***	9.8	4.5	11.7	4.9	9.6	4.9	14.6	5.0			HA4 NS1 NS3 NS4 P
Hypomania personality scale***	8.9	5.6	16.3	7.6	8.4	5.8	9.0	5.9			HA1 NS3
Symptoms of anxiety ***	1.2	0.2	1.3	0.3	1.2	0.2	1.4	0.3			HA2 HA3 HA4 RD1
Symptoms of depression***	1.2	0.3	1.3	0.3	1.3	0.3	1.5	0.4			HA2 HA3 NS3 RD1 RD3
Alexithymia, factor 1***	11.7	5.3	12.0	5.4	12.7	5.2	16.0	5.4			HA2 NS1 RD1 RD3 RD4
Alexithymia, factor 2***	13.5	3.7	12.6	3.2	12.8	3.1	15.3	2.9			HA1 HA2 RD4
Alexithymia, factor 3*	28.6	4.3	28.6	4.0	27.9	3.8	28.5	3.1			

* *p*<0.05 uncorrected.

** *p*<0.05 corrected.

*** *p*<0.01 corrected.

a First scale: the individual TCI scale with the strongest significant association to the variable.

b Second scale: any other individual TCI scale with a significant association to the variable that is stronger than the clusters.

c Other scale(s): any other individual TCI scale that is significantly associated to the variable. For significant associations between clusters and variables from the psychological scales, the mean value for the cluster with the highest scores are in bold. Psychological scales included the Schizoid Scale (from the Minnesota Multiphasic Personality Inventory); *Perceptual Aberration Scale*, *Physical Anhedonia Scale* and *Social Anhedonia Scale; Bipolar II scale*; *Hypomanic Personality Scale*; *Symptoms of anxiety* and *symptoms of depression* from the HSCL-25; and Alexithymia factors 1–3 from the Twenty Item Toronto Alexithymia Scale (Factor 1: difficulties in identifying feelings; Factor 2: difficulties in describing feelings; and Factor 3: externally oriented thinking).

Health-related quality of life was assessed with the 15D measure [20]. The HSCL-25-depression questionnaire, which is a 25-item shortened version of the original 90-item questionnaire [21], was used for measurement of symptoms for depression and anxiety as described by Veijola et al [22], and the Twenty Item Toronto Alexithymia Scale (TAS-20), which has been translated into Finnish [23], was used to measure the three facets of alexithymia [24]. Subjects were asked whether they had ever been diagnosed by a physician as having depression (yes/no), while a diagnosis of schizophrenia was based on validated diagnosis [17] data from hospital discharge register up until the end of year 1997.

The second questionnaire included a Finnish translation of the 107-item *Temperament and Character Inventory* (TCI) for measurement of four dimensions of temperament (NS, HA, RD and P) and their respective subscales (HA1: anticipatory worry, HA2: fear of uncertainty, HA3: shyness, HA4: fatigability; NS1: exploratory excitability, NS2: impulsiveness, NS3: extravagance, NS4: disorderliness; RD1: sentimentality, RD3: attachment, RD4: dependence, and P: persistence). Specifically, this was the Tridimensional Personality Questionnaire (TPQ) subset of the TCI version 9, with 107 binary items. Although the TPQ originally measured three dimensions, these original items were rearranged to calculate summary scores for the revised four dimensions of the TCI (with five items originally contributing to RD now analyzed separately as Persistence, and one of the original RD items now contributing to NS). In addition, the questionnaire included the following: the *Perceptual Aberration Scale (PER)*, *Physical Anhedonia Scale (PAS)* and *Social Anhedonia Scale (SAS)* were included to measure traits which indicate a predisposition toward psychosis [25]; the *Schizoid Scale (SCHD)*, extracted from the Minnesota Multiphasic Personality Inventory, to measure psychotic traits [26]; the *Hypomanic Personality Scale (HPS)* [27]; and a scale referred to here as *“Bipolar II scale”* to identify those depressed subjects who are at risk for later conversion to bipolar disorders [28]. For reference of collection and application of these scales, see Miettunen et al. [29], [30].

All subjects included in the present study gave written consent for their data to be used. Permission to gather registry data was obtained by the Finnish Ministry of Social Welfare and health. The study was approved by the Ethics Committee of the Faculty of Medicine, University of Oulu.

An additional sample was examined in order to replicate the cluster structure identified in the NFBC1966 cohort. In the *Cardiovascular Risk in Young Finns* study (YF), which began in 1980, subjects for the original sample (N = 3,596) were selected randomly from six different age cohorts (3 to 18 years) in the population register of the Social Insurance Institution, a database covering the whole population of Finland. The design of the study and the selection of the sample have been described in detail by Raitakari et al [31]. Measurements for the present study were carried out in year 2001 when 2,105 participants (59% of the 1980 cohort) completed the TCI-version 9 questionnaire. As there was no significant association of the TCI scales to age group in this cohort, we used the TCI variables without any further adjustment for age.

### Data Analysis

In cluster analysis, a set of individuals is divided into groups such that individuals designated to the same group are as similar to each other as possible while being as different from individuals in other groups as possible. Clustering methods applied to psychological data have lead to biologically meaningful results in previous work on schizophrenia by members of our group [32].

A large number of different clustering methods exist, often tailored to specific data types or applications. We performed initial tests with the probabilistic Gaussian mixture model clustering [33], a density-based clustering method [34], and the *k*-means method [35], and chose *k*-means for further analysis. We chose k-means for further analysis, as this method is well established in the literature and, compared to the other two methods, produces very robust and stable clusterings of temperament data, and delivers easily interpretable prototypical descriptions for the clusters found in the form of cluster centers.

Before clustering, all the scales were normalized to mean 0 and SD 1. The Euclidean distance between the 12-dimensional temperament subscale vectors was used as similarity measure. As the distribution of the subscales in the two genders differ significantly [7], [8], clustering and subsequent analyses were performed separately for both genders. The *k*-means clustering algorithm requires the user to select *k*, the number of clusters. We computed clusterings for 2–12 clusters and selected the best model from among those by the Bayesian information criterion [36].

### Replication Analysis

To further analyze the validity of the clusters, we performed a cluster analysis on the TCI temperament subscales in the YF sample. Assigning a cluster to the individuals in the replication sample based on the model obtained on original NFBC1966 data, and vice versa, enabled us to analyze whether these two models represent the same underlying population structure. Further details of this method and results are presented in (Materials S1), including Figure S1 and Tables S1, S2, S3.

### Association Analyses

In order to test differences between clusters on outcome variables representing a range of life domains, we conducted one-way analyses of variance for continuous variables and a chi-square tests of independence for discrete variables. A total of 115 variables representing occupation, lifestyle, socioeconomic status, and mental and physical health were examined. The collection of data in the NFBC1966 is extensive; in order to span the range of adult health and outcome while including those variables with adequate data, we selected variables if 1) sufficient information was available about the nature of the variable (i.e., how information was collected and measured), 2) more than 50% of cohort members had data available for that variable, and 3) we considered that variable to be meaningful for psychological and somatic health. An un-weighted Bonferroni correction [37] for *p*-values was applied to compensate for multiple hypothesis testing.

In order to compare results between temperament clusters and original subscale scores, we also tested differences between each individual subscale and the same variables representing adult outcome across a range of life domains. All individuals were ordered based on their score in the subscale in question and split into four groups of sizes equal to the clusters. For these groups, we performed the same statistical test as was used for the clusters, corrected again with the Bonferroni correction, and compared the strength of the resulting association with that obtained from analyses using the clusters.

## Results

### Cluster Analyses

Clustering of the NFBC1966 gender-specific samples according to the TCI *main* scales did not yield evidence for a cluster structure. In contrast, clustering of the 12 TCI *subscales* showed stable results with an optimum of four clusters in both genders (Figure 1). In the replication of the TCI subscale clustering in the YF sample, Cohen’s kappa values were between 0.7 and 0.9, indicating a strong agreement between the models based on the separate samples (see Materials S1).

Despite genders having been clustered separately, we found similar clusters in the gender-specific models. Figure 2 shows these clusters as star plots (with 0 as sample mean and 1 as sample standard deviation). For females, Clusters I, II, III and IV include 26%, 25%, 28% and 21% of the subjects, whereas for males these numbers are 26%, 22%, 30% and 22%.

### Association Analyses

The number and proportion of variables that are significantly associated with cluster membership and TCI scales are presented in Table 1. We examined the associations of temperament and the *health-related quality of life* as measured with the 15D measure [20] (Tables 2–3). Cluster IV consistently reported the most problems across the 15 dimensions measured, while Clusters I and II reported the least problems both in males and females. Of the subscales, HA-1 and HA-4 had power equal to the clusters to “predict” these variables, with RD-1, RD-3, NS-1, NS-3 and the other HA scales also having some associations, although with less power than the clusters.

We tested for differences in *education, work and socioeconomic status* between the four clusters, and found significant differences in education (Tables S4–S5). For example, only 41% and 26% of women and men in Cluster IV had finished secondary school, while 56% and 35% in Cluster II had finished secondary school. These clusters also represent the two extremes in higher level of education. In concordance, Cluster IV reported lowest working capacity in both genders. Marital status differed between clusters, with the highest rate of marriage in Cluster I for both females (62%) and males (50%), and the highest rate of divorce in Cluster II females (7%) and males (5%). For males, the TCI subscales most strongly associated to these variables were HA-3 and HA-4. For females, there were very few strong associations of the individual scales, with the exception of a strong association of rate of marriage to NS-3 (in females only).

Individuals from Cluster II reported the best, while those from Cluster IV the worst, physical functional capacity both in females and males. Over 10% of Cluster II females, but only 4% of Cluster IV females, reported no trouble running 2 or 5 kilometers, with a similar pattern observed for males. Self-reported physical activity also followed the pattern of self-reported physical functional capacity.

As indicators of taking care of oneself, Cluster IV individuals tended to report brushing their teeth less often than other clusters, while members from Cluster II reported using more alcohol and more members reported smoking regularly (52% of females and 55% males). Almost no statistically significant differences could be observed in the physical measurements of the individuals, including height, weight, BMI, blood pressure, and levels of fasting sugar, insulin, and cholesterol. Triglyceride levels were slightly higher in Cluster IV (1.35 mmol/dL) and lower in Cluster I (1.20 mmol/dL) males. Females, particularly those in Cluster II, had a tendency to underestimate their weight in the postal questionnaire compared to the measurements at the physician’s office.

In terms of the individual temperament dimensions, for females, smoking and alcohol were associated to NS-3 almost exclusively, while the HA scales were associated to self-reported physical capacity. NS-3 and the HA scales also dominated the pattern in males, but the performance of clusters vs. scales was the opposite that seen in females. The lack of associations indicate that using only subscales without clustering would miss the associations to physical activity, frequency of brushing teeth, lifetime abstinence from alcohol, calcium intake, and difference in self-reported and measured BMI in females. In males, while using the scales instead of clusters would miss associations to the brushing of teeth, using the clusters alone would miss associations certain physical variables, all strongly associated to NS-3.

In terms of confirmed *diagnoses*, we found minor differences in physical health, with a considerable difference between males and females (Table 4). For females, individuals in Cluster IV reported less allergic rhinitis and eczemas, and more rheumatoid arthritis than individuals in other clusters. Among males, Cluster II individuals had almost double the prevalence of asthma compared to the other clusters, while individuals in Cluster II and IV had hypertension more often. For both genders, self-reported lifetime-depression and register-based diagnosis of schizophrenia were over twice as common in Cluster IV, while Cluster I clearly had the lowest prevalence. In terms of the individual subscales, depression was associated to HA-4 in both genders, while there were no additional associations between other diagnoses and individual subscales.

We also analyzed associations to certain validated *psychological scales* completed by the participants (Tables 5–6). Individuals from Cluster IV scored the highest and individuals from Cluster I or II the lowest on all scales measuring traits predisposing to psychosis, including the *PER*, *PAS*, *SAS* and *SCHD*. Similar findings were obtained with *SCL and TAS-20* factors, with individuals in Clusters I and II scoring consistently lowest and individuals from Cluster IV highest on symptoms related to anxiety, depression and alexithymia. Interestingly, individuals from Cluster II scored highest on the HPS (mean in females 16.2 and in males 16.4) while individuals from Cluster III scored lowest (mean in females 9.5 and in males 8.5).

These psychological scales were also associated to many of the TCI subscales (Tables 5–6). For example, in females, we found strong associations of PAS to RD-1 and HA-1, and SCL symptoms to HA-1 and HA-4. It is interesting to note that while for males the HA scales dominate the association picture, there are two scales that were not associated to any temperament dimension: the schizoid scale, and the social anhedonia scale. In addition, in females, the cluster association to the HPS would be missed by an analysis using the subscales alone.

## Discussion

We present a stable and robust clustering of domains of temperament in a population-based sample. Our results on >2,000 females and >1,700 males from a longitudinal birth cohort from Northern Finland demonstrate that the analyzed temperament dimensions of the TCI do not distribute randomly among individuals but have a consistent, endogenous pattern, and this structure is supported by a replication analysis in a separate, representative population sample of >2,000 Finnish individuals. In addition, our results provide further evidence for the importance of temperament to health and well being, with statistically significant differences between these temperament clusters across a number of life domains.

### Properties of the Temperament Clusters

A stable and robust clustering was found with the TCI *subscales* with an optimum of four clusters (I-IV) for both males and females. The results proved to be quite similar for both genders, despite the separate analyses, and in agreement in a replication sample, providing further support for the stability of these temperament profiles. We did not, however, find evidence for a stable clustering pattern based on the four TCI scales alone; this is likely because the use of subscales provided more information that the clustering algorithm could use to partition the individuals into stable and robust clusters.

Individuals from *Cluster I* can be described as stable, persistent and not very impulsive. They report a high quality of life and self-reported working capacity, and a relatively high level of education. Both females and males are more often married than individuals from the other clusters. Their life habits are healthy: they brush their teeth, do not drink very much alcohol and only rarely smoke. They score lowest in most of the scales for psychosis proneness and symptoms for depression and anxiety, and this cluster has a lower prevalence of depression and schizophrenia than other clusters. Consequently, our results suggest that this temperament profile, which is characterized by remarkably average levels on most of the temperament traits except particularly low levels of impulsivity (HA2) and disorderliness (HA4), may possess features enabling mental stability and psychological adaptability, leading to practice of healthy life habits, stable life features, and decreased risk for mental disorders.

Individuals from *Cluster II* can be characterized as outgoing, energetic people who tend to be impulsive. Like individuals from Cluster I, they have a high quality of life and self-reported working capacity. They have the highest level of education, their annual income is on average higher than other clusters, and they also report the best physical functional capacity. Divorces are more common and they have a tendency for higher consumption of alcohol and more smoking, particularly in females. Cluster II is characterized by relatively low levels of depression and schizophrenia, supported by low scores for all psychological scales that measure traits predisposing toward psychosis, anxiety or depression, except on the hypomania personality scale, on which Cluster II members score the highest. It is noteworthy that we observed here a tendency of Cluster II individuals to embellish reality. This is consistent with this cluster’s temperament profile, as well as the high scores on the HPS [38]. Individuals with hypomanic personality have been reported to provide high estimates of their future academic and occupational performance [27], [38], which leads to a need for caution when interpreting the self-reports of positive lifestyle and health-related variables in Cluster II.

In terms of quality of life, socioeconomic status, mental and physical health, individuals from *Cluster III* do not show any extreme characteristics. The education, working capacity and physical functional capacity are higher in Cluster III than in Cluster IV but generally lower than for individuals from Clusters I or II. They score low in scales for psychosis proneness as well as for the hypomania personality scale, the latter being likely to reflect the low energy level of these individuals.

Individuals from *Cluster IV* could be described as shy and pessimistic persons who prefer routine and privacy. They score the lowest in most fields of health and well-being and are more often unemployed. They also report lowest working capacity scores and their annual income is lowest among the four clusters. Members of Cluster IV are the least physically fit. Virtually all indicators for psychological health show signs for increased mental health problems, both in levels symptoms as well as manifest disorders. Thus, based on the particular traits measured in the NFBC1966, our results suggest that a profile characterized by excessively high HA and low NS, RD and P (representing approximately 20% of cohort members) may capture a profile of increased physical and mental health risk.

Overall, our results are in line with the previously published findings. In a previous study on NFBC1966, all four domains of temperament were found to be associated to socioeconomic status, alcohol consumption and smoking behavior in varying configurations [10]. There was a negative gradient between HA and level of education and a tendency towards higher RD and P with increasing socioeconomic status. Previous studies have also demonstrated relationships between high HA (and other related personality traits, such as high Neuroticism and dysthymic temperament) with depression and anxiety [39], [40], [41], [42], [43]. In our current analyses, we also found that the clusters do as well as, and in many cases better than, the individual dimensions to find associations with outcome variables. A further advantage of using these clusters to investigate the relationship between temperament and health outcomes is that clusters have an additional value of simpler data structure (as each individual has only one definition), meaning also that fewer statistical tests are needed.

### Strengths and Limitations

The primary strength of our analyses stems from the prospective design of the study and follow-up of a large birth cohort, which allows for control of recall bias. Furthermore, a previous analysis has demonstrated that participation in this cohort does not vary across specific disorders, nor do gender or education explain the association of psychiatric disorders with participation [19]. An additional strength of the present study is the relative homogeneity of its population: all subjects were of the same age and ethnicity. This implication of this is that the TCI scores in young Finns are likely not biased by cross-cultural issues associated with temperament measurement [28], [29].

One potential limitation is that temperament in the NFBC1966 was assessed only at one time point. However, although absolute scores in temperament may change over time, inter-individual differences typically remain relatively stable [42]. Nevertheless, application of repeated measures of temperament would likely add to the accuracy of the results.

A final limitation is that clustering itself cannot answer the question of whether the clusters found reflect real clusters in the data or are artifacts of the method. However, to begin to address this limitation, we replicated the clustering analysis in a separate sample that represents the Finnish population well, thereby providing additional support for this pattern of temperament profiles.

### Implications

To our knowledge, this is the first study to investigate temperament patterns using cluster analysis tools in population-based samples of both females and males. One previous study reported an association between temperament profiles and a high level of physiological CHD risk factors; however, this study included only men and was comprised of a relatively small sample of 190 individuals [44].

Our results further question the assumption that temperament domains are independent. The heritability of each temperament factor has been estimated to range from 50 to 65% [2], [3], [4], [5]. Although these factors have been suggested to be independent of each other, contradictory results have also been reported [45]. For example, a recent meta-analysis supports temperament dimension inter-relatedness, particularly given the consistent negative correlation between NS and HA in a number of studies [3]. These results also lend support to a person-centered approach. While the majority of studies to combine independent dimensions and examine the ability of resulting temperament profiles to predict mental health have focused on samples of children and adolescents [46], [47], [48], [49], [50], our results support the argument that additional information can be obtained by considering cluster profiles. Indeed, in a manuscript submitted concurrently, we demonstrate significant relationships between these same cluster profiles and life course measures (e.g., early environment, neurobehavioral development, and adolescent behavior)(Congdon et al., submitted concurrently).

Obtaining a better understanding of these relationships will be critical for understanding the underlying genetic architecture and other possible etiological processes predisposing individuals to a particular temperament profiles, as well as the relationship between genetic factors, patterns of temperament, and ultimately psychiatric and somatic health outcomes.

## Supporting Information

Figure S1 **Histograms of chi-square values.** Chi-square values of 100 experiments using generated data and of results based on cross-tabulation of 4-cluster solutions (presented in Table S2), with green for females and red for males.
(TIF)

Table S1 **Clusterings based on NFBC66 four-cluster model vs. YF two-cluster mode.**
(DOC)

Table S2 **Clusterings based on NFBC66 four-cluster model vs. YF four-cluster mode.**
(DOC)

Table S3 **Clusterings based on NFBC66 two-cluster model vs. YF two-cluster mode.**
(DOC)

Table S4 **Self-rated physical capacity, life habits, health and stress reactivity data from the 31-year follow-up in NFBC66 females.**
(DOC)

Table S5 **Self-rated physical capacity, life habits, health and stress reactivity data from the 31-year follow-up in NFBC66 males.**
(DOC)

Materials S1 (DOC)

## References

[1] Oliver JP, Robins RW, Pervin LA (2008). Handbook of Personality, Third Edition: Theory and Research.. New York: The Guilford Press.

[2] Ando J, Ono Y, Yoshimura K, Onoda N, Shinohara M (2002). The genetic structure of Cloninger’s seven-factor model of temperament and character in a Japanese sample.. Journal of Personality.

[3] Gillespie NA, Cloninger CR, Heath AC, Martin NG (2003). The genetic and environmental relationship between Cloninger’s dimensions of temperament and character.. Personality and Individual Differences.

[4] Heath AC, Cloninger CR, Martin NG (1994). Testing a model for the genetic structure of personality: A comparison of the personality systems of Cloninger and Eysenck.. J Pers Soc Psychol.

[5] Stallings MC, Hewitt JK, Cloninger CR, Heath AC, Eaves LJ (1996). Genetic and environmental structure of the Tridimensional Personality Questionnaire: Three or four temperament dimensions?. J Pers Soc Psychol.

[6] Cloninger CR (1987). A systematic method for clinical description and classification of personality variants. A proposal.. Archives of General Psychiatry.

[7] Cloninger CR, Przybeck TR, Švrakić DM, Wetzel RD (1994). The Temperament and Character Inventory (TCI): A Guide to Its Development and Use. St.. Louis: Washington University, Center for Psychobiology of Personality.

[8] Miettunen J, Veijola J, Lauronen E, Kantojärvi L, Joukamaa M (2007). Sex differences in Cloninger’s temperament dimensions – a meta-analysis.. Comprehensive Psychiatry.

[9] Smith TW, MacKenzie J (2006). Personality and risk of physical illness.. Annu Rev Clin Psychol.

[10] Sovio U, King V, Miettunen J, Ek E, Laitinen J (2007). Cloninger’s Temperament dimensions, socio-economic and lifestyle factors and metabolic syndrome markers at age 31 years in the Northern Finland Birth Cohort 1966.. J Health Psychol.

[11] Battaglia M, Przybeck TR, Bellodi L, Cloninger CR (1996). Temperament dimensions explain the comorbidity of psychiatric disorders.. Comprehensive Psychiatry.

[12] Engström C, Brändström S, Sigvardsson S, Cloninger R, Nylander PO (2004). Bipolar disorder: I. Temperament and character.. Journal of Affective Disorders.

[13] Fassino S, Abbate-Daga G, Amianto F, Leombruni P, Boggio S (2002). Temperament and character profile of eating disorders: A controlled study with the temperament and character inventory.. International Journal of Eating Disorders.

[14] Klump KL, Strober M, Bulik CM, Thornton L, Johnson C (2004). Personality characteristics of women before and after recovery from an eating disorder.. Psychological Medicine.

[15] Ritsner M, Susser E (2004). Temperament types are associated with weak self-construct, elevated distress and emotion-oriented coping in schizophrenia: Evidence for a complex vulnerability marker?. Psychiatry Research.

[16] Miettunen J, Veijola J, Lauronen E, Kantojärvi L, Joukamaa M (2008). Inter-correlations between Cloninger’s temperament dimensions – a meta-analysis.. Psychiatry Research.

[17] Rantakallio P (1969). Groups at risk in low birth weight infants and perinatal mortality.. Acta Paediatrica Scandinavica.

[18] Moilanen K, Veijola J, Läksy K, Mäkikyrö T, Miettunen J (2003). Reasons for the diagnostic discordance between clinicians and researchers in schizophrenia in the Northern Finland 1966 Birth Cohort.. Social Psychiatry and Psychiatric Epidemiology.

[19] Haapea M, Miettunen J, Läärä E, Joukamaa MI, Järvelin MR (2008). Non-participation in a field survey with respect to psychiatric disorders.. Scand J Public Health.

[20] Sintonen H, Pekurinen M (1989). A generic 15 dimensional measure of health-related quality of life (1SD).. J Soc Med.

[21] Derogatis LR, Lipman RS, Covi L (1973). SCL-90: An outpatient psychiatric rating scale–preliminary report.. Psychopharmacological Bulletin.

[22] Veijola J, Jokelainen J, Läksy K, Kantojärvi L, Kokkonen P (2003). The Hopkins Symptom Checklist-25 in screening DSM-III-R axis-I disorders.. Nordic Journal of Psychiatry.

[23] Joukamaa M, Miettunen J, Kokkonen P, Koskinen M, Julkunen J (2001). Psychometric properties of the Finnish 20-item Toronto Alexithymia Scale.. Nordic Journal of Psychiatry.

[24] Bagby RM, Parker JDA, Taylor GJ (1994). The twenty-item Toronto Alexithymia Scale-I. Item selection and cross-validation of the factor structure.. Journal of Psychosom Res.

[25] Chapman LJ, Chapman JP, Raulin ML (1976). Scales for physical and social anhedonia.. Journal of Abnormal Psychology.

[26] Golden RR, Meehl P (1979). Detection of the schizoid taxon with MMPI indicators.. Journal of Abnormal Psychology.

[27] Eckblad M, Chapman LJ (1986). Development and validation of a scale for hypomanic personality.. Journal of Abnormal Psychology.

[28] Akiskal HS, Maser JD, Zeller PJ, Endicott J, Coryell W (1995). Switching from ‘unipolar’ to bipolar II. An 11-year prospective study of clinical and temperamental predictors in 559 patients.. Archives of General Psychiatry.

[29] Miettunen J, Kantojärvi L, Ekelund J, Veijola J, Karvonen JT (2004). A large population cohort provides normative data for investigation of temperament.. Acta Psychiatrica Scandinavica.

[30] Miettunen J, Veijola J, Freimer N, Lichtermann D, Peltonen L (2008). Data on schizotypy and affective scales are gender and education dependent – study in the Northern Finland 1966 Birth Cohort.. Psychiatry Research.

[31] Raitakari OT, Juonala M, Rönnemaa T, Keltikangas-Järvinen L, Räsänen L (2008). Cohort profile: The Cardiovascular Risk in Young Finns Study.. International Journal of Epidemiology.

[32] Wessman J, Paunio T, Tuulio-Henriksson A, Koivisto M, Partonen T (2009). Mixture model clustering of phenotype features reveals evidence for association of DTNBP1 to a specific subtype of schizophrenia.. Biological Psychiatry.

[33] McLachlan G, Peel D (2000). Finite Mixture Models.. Hoboken, NJ: John Wiley & Sons, Inc.

[34] Ester M, Kriegel HP, Sander J, Xu X (1996). A Density-Based Algorithm for Discovering Clusters in Large Spatial Databases with Noise.. Proc 2nd Int Conf on Knowledge Discovery and Data Mining.

[35] MacQueen J (1967). Some methods for classification and analysis of multivariate observations.. Proc 5th Berkeley Symp Math Statist Prob.

[36] Schwarz G (1978). Estimating the dimension of a model.. The Annals of Statistics.

[37] Shaffer JP (1995). Multiple hypothesis testing.. Annu Rev Psychol.

[38] Meyer TD, Krumm-Merabet C (2003). Academic performance and expectations for the future in relation to a vulnerability marker for bipolar disorders: the hypomanic temperament.. Personality and Individual Differences.

[39] Farmer A, Mahmood A, Redman K, Harris T, Sadler S (2003). A sib-pair study of the Temperament and Character Inventory scales in major depression.. Arch Gen Psychiatry.

[40] Elovainio M, Kivimaki M, Puttonen S, Heponiemi T, Pulkki L (2004). Temperament and depressive symptoms: A population-based longitudinal study on Cloninger’s psychobiological temperament model.. J Affect Disord.

[41] Nyman E, Miettunen J, Freimer N, Joukamaa M, Maki P (2011). Impact of temperament on depression and anxiety symptoms and depressive disorder in a population-based birth cohort.. J Affect Disord.

[42] Cloninger CR, Švrakić DM, Przybeck TR (2006). Can personality assessment predict future depression? A twelve-month follow-up of 631 subjects.. Journal of Affective Disorders.

[43] Jylha P, Isometsa E (2006). Temperament, character and symptoms of anxiety and depression in the general population.. Eur Psychiatry.

[44] Keltikangas-Järvinen L, Ravaja N, Viikari J (1999). Identifying Cloninger’s temperament profiles as related to the early development of the metabolic cardiovascular syndrome in young men.. Arterioscler Thromb Vasc Biol.

[45] Herbst JH, Zonderman AB, Mccrae RR, Costa PT (2000). Do the dimensions of the temperament and character inventory map a simple genetic architecture? Evidence from molecular genetics and factor analysis.. American Journal of Psychiatry.

[46] Janson H, Mathiesen KS (1008). Temperament profiles from infancy to middle childhood: development and associations with behavior problems.. Developmental Psychology.

[47] Komsi N, Räikkönen K, Pesonen AK, Heinonen K, Keskivaara P (2006). Continuity of temperament from infancy to middle childhood.. Infant Behav Dev.

[48] Caspi A, Moffitt TE, Newman DL, Silva P (1996). Behavioral observations at age 3 years predict adult psychiatric disorders. Longitudinal evidence from a birth cohort.. Archives of General Psychiatry.

[49] Crockett LJ, Moilanen KL, Raffaelli M, Randall BA (2006). Psychological profiles and adolescent adjustment: A person-centered approach.. Development and Psychopathology.

[50] Rettew DC, Althoff RR, Dumenci L, Ayer L, Hudziak JJ (1008). Latent profiles of temperament and their relations to psychopathology and wellness.. Journal of the American Academy of Child & Adolescent Psychiatry.

