# Supplementary figures and images for: Temperament Clusters in a Normal Population: Implications for Health and Disease
Source: PLoS One. 2012 Jul 18;7(7):e33088. doi: 10.1371/journal.pone.0033088 (PMC3399883; doi:10.1371/journal.pone.0033088)

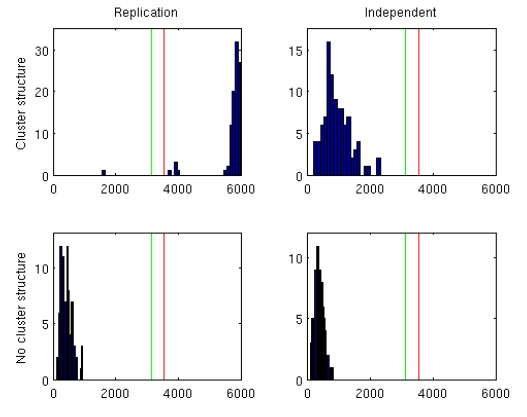

Supplement: Figure S1 — Histograms of chi-square values. Chi-square values of 100 experiments using generated data and of results based on cross-tabulation of 4-cluster solutions (presented in Table S2), with green for females and red for males. (TIF) [file pone.0033088.s001.tif]
