# Supplementary material for: Temperament Clusters in a Normal Population: Implications for Health and Disease
Source: PLoS One. 2012 Jul 18;7(7):e33088. doi: 10.1371/journal.pone.0033088 (PMC3399883; doi:10.1371/journal.pone.0033088)
Supplement: Table S1 — Clusterings based on NFBC66 four-cluster model vs. YF two-cluster mode. (DOC) [file pone.0033088.s002.doc]

Table S1. Clusterings based on NFBC66 four-cluster model vs. YF two-cluster mode

|  | Both datasets | | YF dataset only | | NFBC66 only | |
| --- | --- | --- | --- | --- | --- | --- |
| **NFBC66 Cluster** | **YF 1** | **YF 2** | **YF 1** | **YF 2** | **YF 1** | **YF 2** |
| Female Cluster I | *514* | 336 | *189* | 124 | *325* | 212 |
| Female Cluster II | *739* | 17 | *245* | 11 | *494* | 6 |
| Female Cluster III | 208 | *447* | 23 | *67* | 185 | *380* |
| Female Cluster IV | 0 | *657* | 0 | *224* | 0 | *433* |
| Male Cluster I | 168 | *495* | 64 | *150* | 104 | *345* |
| Male Cluster II | 0 | *631* | 0 | *247* | 0 | *384* |
| Male Cluster III | 421 | *566* | 208 | *266* | 213 | *300* |
| Male Cluster IV | *659* | ***0*** | *279* | 0 | *380* | 0 |

Cells in italics represent the highest agreement of individuals between the cluster solutions being compared.
