# Supplementary material for: Temperament Clusters in a Normal Population: Implications for Health and Disease
Source: PLoS One. 2012 Jul 18;7(7):e33088. doi: 10.1371/journal.pone.0033088 (PMC3399883; doi:10.1371/journal.pone.0033088)
Supplement: Table S3 — Clusterings based on NFBC66 two-cluster model vs. YF two-cluster mode. (DOC) [file pone.0033088.s004.doc]

Table S3. Clusterings based on NFBC66 two-cluster model vs. YF two-cluster mode

|  | Both datasets | | YF dataset only | | NFBC66 only | |
| --- | --- | --- | --- | --- | --- | --- |
| **NFBC66 Cluster** | **YF 1** | **YF 2** | **YF 1** | **YF 2** | **YF 1** | **YF 2** |
| Female Cluster I | 0 | *1262* | 0 | *361* | 0 | *901* |
| Female Cluster II | *1461* | 195 | *457* | 65 | *1004* | 130 |
| Male Cluster I | 12 | *1591* | 6 | *620* | 6 | *971* |
| Male Cluster II | *1236* | 101 | *545* | 43 | *691* | 58 |

Cells in italics represent the highest agreement of individuals between the cluster solutions being compared. Cohen’s kappa values for agreement between the four-cluster solutions based on data from both datasets, the YF dataset only, and the NFBC66 dataset only for females are 0.87, 0.85, and 0.87, respectively, and for males are 0.92, 0.91, and 0.92, respectively.
