# Supplementary material for: Temperament Clusters in a Normal Population: Implications for Health and Disease
Source: PLoS One. 2012 Jul 18;7(7):e33088. doi: 10.1371/journal.pone.0033088 (PMC3399883; doi:10.1371/journal.pone.0033088)
Supplement: Table S5 — Self-rated physical capacity, life habits, health and stress reactivity data from the 31-year follow-up in NFBC66 males. (DOC) [file pone.0033088.s006.doc]

Table S5. Self-rated physical capacity, life habits, health and stress reactivity data from the 31-year follow-up in NFBC66 males.

|  | **Clusters** | | | | | | | | **Subscales** | | |
| --- | --- | --- | --- | --- | --- | --- | --- | --- | --- | --- | --- |
|  | **I** | | **II** | | **III** | | **IV** | | **First^a^** | **Second^b^** | **Other^c^** |
|  | **Prop** |  | **Prop** |  | **Prop** |  | **Prop** |  |  |  |  |
| *Education**** |  |  |  |  |  |  |  |  |  |  |  |
| Basic education: comprehensive school | 69% |  | 64% |  | 69% |  | 73% |  | HA3 |  |  |
| Basic education: secondary school* | 31% |  | **35%** |  | 31% |  | 26% |  | HA3 |  |  |
| Further education: none*** | 3.4% |  | 5.0% |  | 5.9% |  | **11%** |  |  |  |  |
| Further education: vocational secondary | 44% |  | 41% |  | 49% |  | 49% |  | HA3 |  |  |
| Further education: college education* | 32% |  | **33%** |  | 28% |  | 24% |  |  |  |  |
| Further education: academic degree* | 12% |  | 12% |  | 7.7% |  | 7.5% |  |  |  |  |
| *Socioeconomic status**** |  |  |  |  |  |  |  |  |  |  |  |
| Farmer | 4.3% |  | 3.9% |  | 4.1% |  | 5.6% |  |  |  |  |
| Other entrepreneur* | 12% |  | 12% |  | 7.7% |  | 6.4% |  |  |  |  |
| Upper white-collar** | 19% |  | **25%** |  | 17% |  | 13% |  | HA3 | HA1 |  |
| Lower white collar | 19% |  | 21% |  | 18% |  | 14% |  |  |  |  |
| Blue collar* | 36% |  | 30% |  | 38% |  | **40%** |  | HA3 |  |  |
| Student* | 2.9% |  | 1.3% |  | **4.7%** |  | 3.5% |  |  |  |  |
| Pensioner | 0.2% |  | 0.3% |  | 0.6% |  | 1.3% |  |  |  |  |
| Unemployed*** | 5.4% |  | 5.8% |  | 8.9% |  | **15.2%** |  |  |  | HA4 |
| Other | 0.7% |  | 1.6% |  | 0.6% |  | 0.5% |  |  |  |  |
| *Marital status**** |  |  |  |  |  |  |  |  |  |  |  |
| Married*** | **50%** |  | 44% |  | 46% |  | 33% |  |  |  |  |
| Cohabitant | 25% |  | 26% |  | 25% |  | 22% |  |  |  |  |
| Unmarried*** | 24% |  | 25% |  | 27% |  | **43%** |  |  |  | HA1 HA2 HA4 RD3 |
| Divorced of separated* | 2% |  | **5%** |  | 2% |  | 2% |  |  |  |  |
| *Self-rated physical functioning capacity and life habits* |  |  |  |  |  |  |  |  |  |  |  |
| Can run 5 km without problems*** | 31% |  | **34%** |  | 26% |  | 20% |  | HA4 |  | HA3 |
| Can run 2 km without problems*** | 62% |  | **64%** |  | 56% |  | 43% |  |  |  | HA3 HA4 RD3 |
| Can lift and carry heavy objects without problems*** | **91%** |  | 90% |  | 85% |  | 78% |  | HA4 |  | HA2 HA3 NS1 |
| Physical activity (active or very active) ** | 41% |  | **49%** |  | 41% |  | 38% |  |  |  |  |
| Brushes teeth more than once a day*** | 38% |  | **47%** |  | 39% |  | 28% |  |  |  |  |
| Brushes teeth about once a day* | 52% |  | 44% |  | 52% |  | **59%** |  |  |  |  |
| Seldom brushes teeth | 10% |  | 9% |  | 9% |  | 14% |  |  |  |  |
| *Alcohol consumption and smoking* |  |  |  |  |  |  |  |  |  |  |  |
| Never used alcohol | 5.1% |  | 5.0% |  | 5.7% |  | 7.2% |  |  |  |  |
| Used to drink but has stopped | 2.2% |  | 0.5% |  | 1.4% |  | 2.9% |  |  |  |  |
| Uses alcohol less than once a month | 15% |  | 12% |  | 12% |  | 15% |  | NS3 |  |  |
| Uses alcohol at least once a month* | 77% |  | **82%** |  | 81% |  | 75% |  | NS3 |  |  |
| Has smoked at least once in lifetime** | 57% |  | 68% |  | **70%** |  | 63% |  | NS3 |  |  |
| Smokes regularly** | 45% |  | 56% |  | **59%** |  | 51% |  | NS3 |  |  |
| Smokes every day | 19% |  | 30% |  | 32% |  | 23% |  | NS3 |  |  |
| Smokes almost every day | 1.1% |  | 3.7% |  | 3.4% |  | 2.4% |  |  |  |  |
| Smokes 2-4 times a week | 2.2% |  | 3.2% |  | 2.8% |  | 1.6% |  |  |  |  |
| Smokes once a week | 0.9% |  | 0.0% |  | 1.0% |  | 0.3% |  |  |  |  |
| Smokes only occasionally | 11% |  | 9.2% |  | 11% |  | 12% |  |  |  |  |
| Never smokes these days*** | **66%** |  | 54% |  | 50% |  | 60% |  | NS3 |  |  |
| *Working capacity, self reported* | **Mean** | **SD** | **Mean** | **SD** | **Mean** | **SD** | **Mean** | **SD** | **BestS** | **OBS** | **OSS** |
| General*** | 9.1 | 1.0 | **9.0** | 1.3 | 8.8 | 1.3 | 8.2 | 1.7 |  |  | HA1 HA2 HA3 NS1 RD1 RD3 |
| Physical*** | 4.5 | 0.7 | **4.5** | 0.8 | 4.3 | 0.8 | 4.2 | 0.8 | HA4 |  | HA1 HA2 |
| Mental*** | 4.5 | 0.6 | **4.6** | 0.6 | 4.4 | 0.7 | 4.0 | 0.8 |  |  | HA2 NS1 RD3 |
| Illness/handicap | 5.8 | 0.7 | 5.8 | 0.8 | 5.9 | 0.8 | 5.8 | 1.1 |  |  |  |
| Absences from work* | 4.4 | 0.7 | 4.3 | 0.8 | 4.4 | 0.7 | 4.4 | 0.8 |  |  |  |
| Health in the future*** | **6.9** | 0.7 | 6.8 | 1.0 | 6.8 | 0.9 | 6.5 | 1.3 | HA4 |  | NS1 |
| Enjoying daily activities*** | 3.3 | 0.8 | 3.3 | 0.8 | 3.1 | 0.9 | 2.7 | 0.9 |  |  | HA2 HA3 HA4 NS1 RD3 |
| Activity and energy*** | 3.0 | 0.6 | 3.0 | 0.7 | 2.8 | 0.7 | 2.4 | 0.8 |  |  | HA2 NS1 P RD3 |
| Feeling hopeful*** | 3.0 | 0.7 | **3.1** | 0.8 | 2.7 | 0.8 | 2.2 | 0.9 |  |  | P |
| Annual income of the household (in FIM)** | 171,037 | 76,586 | **180,185** | 86,859 | 164,086 | 81,368 | 152,583 | 94,294 | HA1 | HA2 HA4 |  |
| *Alcohol consumption and smoking* |  |  |  |  |  |  |  |  |  |  |  |
| Alcohol consumption g/d | 10.0 | 13.0 | 16.0 | 24.6 | 13.4 | 15.7 | 14.5 | 28.3 | NS3 | NS4 |  |
| *Physical health and stress reactivity* |  |  |  |  |  |  |  |  |  |  |  |
| Systolic blood pressure (1) | 130.5 | 12.4 | 130.5 | 13.2 | 131.1 | 13.6 | 132.0 | 14.1 |  |  |  |
| Diastolic blood pressure (1) | 80.6 | 11.3 | 80.3 | 11.5 | 80.8 | 11.8 | 81.1 | 12.3 |  |  |  |
| Systolic blood pressure (2) | 129.5 | 12.3 | 129.7 | 12.6 | 129.9 | 13.5 | 131.1 | 14.3 |  |  |  |
| Diastolic blood pressure (2) | 79.5 | 11.4 | 79.2 | 11.5 | 79.5 | 11.7 | 79.9 | 12.5 |  |  |  |
| Pulse in 30 s | 33.3 | 5.4 | 33.6 | 5.8 | 33.7 | 5.9 | 34.2 | 6.0 |  |  |  |
| Height (cm) | 178.1 | 6.1 | 178.9 | 6.5 | 178.3 | 5.9 | 177.4 | 6.7 |  |  |  |
| Weight (kg) | 79.8 | 11.8 | 81.0 | 12.7 | 79.9 | 12.9 | 78.4 | 12.3 |  |  |  |
| Pelvis circumference (cm) | 97.3 | 6.6 | 97.8 | 6.8 | 97.3 | 6.5 | 96.8 | 6.6 |  |  |  |
| Waist circumference (cm) | 88.4 | 9.3 | 88.8 | 9.6 | 88.7 | 10.0 | 88.1 | 9.9 | NS3 |  |  |
| B-Leuk (10e9/l) | 5.6 | 1.5 | 6.0 | 1.7 | 5.8 | 1.6 | 5.8 | 1.6 |  |  |  |
| B-Hb g/l | 152.2 | 9.2 | 151.6 | 9.3 | 151.6 | 8.8 | 152.0 | 8.8 |  |  |  |
| E-MCV | 89.7 | 4.1 | 90.0 | 4.1 | 90.0 | 4.1 | 90.1 | 4.1 |  |  |  |
| Total cholesterol | 5.2 | 1.0 | 5.1 | 1.0 | 5.2 | 1.0 | 5.3 | 1.0 |  |  |  |
| LDL-cholesterol | 3.2 | 0.9 | 3.1 | 0.9 | 3.2 | 0.9 | 3.3 | 0.9 |  |  |  |
| HDL-cholesterol | 1.4 | 0.3 | 1.4 | 0.3 | 1.4 | 0.3 | 1.4 | 0.3 |  |  |  |
| Triglyceride | 1.2 | 0.7 | 1.3 | 0.8 | 1.3 | 0.8 | 1.4 | 0.9 | NS3 |  |  |
| Glucose | 5.1 | 0.5 | 5.1 | 0.5 | 5.2 | 0.6 | 5.3 | 1.3 |  |  |  |
| Insulin | 8.5 | 3.9 | 8.4 | 3.9 | 8.9 | 5.9 | 8.5 | 3.3 |  |  |  |
| Calcium (mg/day) | 1271.4 | 646.1 | 1295.5 | 698.0 | 1205.2 | 726.1 | 1263.2 | 743.8 |  |  |  |
| BMI (postal questionnaire) | 25.1 | 3.2 | 25.2 | 3.3 | 25.0 | 3.6 | 25.0 | 3.6 |  |  |  |
| BMI (examined) | 25.1 | 3.3 | 25.3 | 3.4 | 25.1 | 3.8 | 24.9 | 3.7 | NS3 |  |  |
| Difference between systolic pressure | 1.1 | 5.1 | 0.9 | 5.7 | 1.2 | 5.6 | 0.9 | 5.6 |  |  |  |
| Difference between diastolic pressure | 1.0 | 6.3 | 1.1 | 5.7 | 1.3 | 6.1 | 1.2 | 4.9 |  |  |  |
| Difference between BMI values | 0.0 | 0.9 | -0.1 | 0.9 | 0.0 | 0.8 | 0.1 | 1.1 |  |  |  |

**p* < 0.05 uncorrected, ***p* < 0.05 corrected. ****p* < 0.01 corrected. ^a^First scale: the individual TCI scale with the strongest significant association to the variable; ^b^Second scale: any other individual TCI scale with a significant association to the variable that is stronger than the clusters; ^c^Other scale(s): any other individual TCI scale that is significantly associated to the variable. For significant associations between clusters and variables, the cluster with the highest proportion or the mean value for the cluster with the highest scores are in bold.
