# Supplementary material for: Temperament Clusters in a Normal Population: Implications for Health and Disease
Source: PLoS One. 2012 Jul 18;7(7):e33088. doi: 10.1371/journal.pone.0033088 (PMC3399883; doi:10.1371/journal.pone.0033088)
Supplement: Materials S1 — (DOC) [file pone.0033088.s007.doc]

**Supplementary Materials**

Wessman et al., Temperament clusters in a normal population: Implications for health and disease

**Supplementary Methods**

We first conducted a cluster-based analysis of responses on the TCI in 3,761 mean and women from the *Northern Finland Birth Cohort 1966* (NFBC66), which is reported in the main text. In order to test the stability of these clusters in a separate sample, we also replicated the results of the cluster structure analysis among 2,097 participants of the Cardiovascular Risk in Young Finns study (YF). Our approach to cluster replication in the YF dataset was that if a clustering of a sample represents the true structure in the population that the sample is based on, then one should be able to build a clustering model on another sample of the same population and find that the cluster classifications of individuals in both sample on the two models (built on separate samples) closely resemble each other. We replicated our cluster structure analysis using the YF dataset and were able to obtain a convincing replication of the clustering structure, with Cohen’s kappa values of 0.7 and above.

**Supplementary Data**

Data from 3,596 individuals was available from the YF dataset. Individuals in the YF dataset completed the revised version of the TCI, which scores items on a scale of 1-5, while TCI data in the NFBC66 cohort is binary. However, by normalizing subscales to mean 0 and SD 1, we ensure that the TCI subscale scores between YF and NFBC66 datasets are comparable.

In the YF dataset, 2,097 individuals had the majority of TCI item data (5 or less items missing, with 1,068 individuals with complete TCI item data). The remainder of subjects in the YF dataset had higher rate of missingness, with 1,494 subjects with over 35 items missing. The 2,097 individuals with complete or near-complete data were included for analysis.

**Supplementary Data Analysis**

For clustering, we used the data based on the 12 TCI subscales as described in the main text, and applied the same scripts as used for clustering the NFBC66 data. As in the main text, cluster analyses were conduced on males and females separately. We cross-tabulated individuals (either all individuals or in each dataset separately) by cluster solutions in order to examine agreement between cluster solutions, and calculated Cohen’s kappa as an indication of agreement between cluster solutions.

*Simulations*

In order to examine whether the cross-tabulation of individuals by cluster solutions is indicative of existing, replicated cluster structure, we performed simulations. We generated artificial data from 1) a distribution without cluster structure (a 12-dimensional multivariate normal distribution) and 2) a distribution with cluster structure (a mixture model of four multivariate Gaussians). For each distribution, two samples (“original” and “replication”) were generated. In addition, we generated a third (“independent”) sample from another mixture of multivariate Gaussians with 3-5 centers.

These simulated data were then clustered. By re-clustering the original data with both the replication and independent models, and the replication and independent data with the original model, we were able to determine the chi-square values between original and independent clusters, and between original and replication clusters, under both an existing and non-existent real cluster structure. Experiments were performed for N = 1000 for each sample (N = 2000 total for each chi-square calculated), with 100 separate experiments of each kind. In cases where there was no cluster structure, the number of clusters was artificially forced to an arbitrary k for comparison. For cases where a clustering structure exists, the best k according to the Bayesian information criterion on the original data was used.

**Supplementary Results**

According to the Bayesian information criterion, the best number of clusters based on the YF data is two for both genders. We present results corresponding to this two-cluster and a four-cluster solution in order to match results in the NFBC66.

*Cluster Comparisons*

As scores have been normalized to a mean of 0 and SD of 1 in both datasets, we were able to assign a cluster membership to individuals from one dataset based on the model built using the other dataset. Doing this, we were able to obtain four separate clusterings of all individuals: 1) two cluster solution based on NFBC66 model; 2) four cluster solution based on NFBC66 model; 3) two cluster solution based on YF model; and 4) four cluster solution based on YF model. We compare these clusterings by cross-tabulating individuals (either all individuals, or one dataset at a time) and, as can bee seen in Tables S1-S3, the original clusters have a corresponding majority cluster in the new cluster solutions. Clusterings based on the NFBC66 four-cluster model (best model) vs. YF four-cluster model show the highest agreement (Cohen’s kappa = 0.70 for females and 0.73 for males).

*Simulations*

We simulated data and conducted a series of cluster analyses in order to examine whether results based on the NFBC66 and YF datasets are indicative of an existing, replicated cluster structure. Figure S1 shows a histogram of chi-square values over 100 experiments, indicating that replication in a new sample from the same distribution works quite well; that is, chi-square values are close to the theoretical maximum with only a few exceptions. Replication with an arbitrary, forced k (k = 4 presented in Figure S1) results in even lower chi-square values than one would expect from random clustering (the independent sample vs. the existing cluster structure in the original case is close to the true chi-square distribution).

Figure S1 illustrates the chi-square values of the above cross-tabulation (4x4) tables scales to sum to a total of 2000 (in order to make them compatible with the simulated Ns), with green for females and red for males. As the samples are not completely equivalents (e.g., age and recruitment differences), we did not expect a replication that is as good as between two samples from an identical mixture of multivariate Gaussians. However, we can see that both values are well away from the ranges that one would expect for a sample in which no cluster structure existed, as well as what one would expect from two completely independent samples.

**Supplementary Results Conclusion**

Constructing a clustering model on the YF dataset and comparing it to that built on the NFBC66 dataset reveals that the models are remarkably similar. The cluster centers fall close to each other and when comparing the cluster labels of individuals based on difference models, we find high agreement (Cohen’s kappa values around 0.70 and above). Our simulation analyses also reveal chi-square values that are clearly out of the range experimentally seen for random clusterings, or clusterings of population without structure. Based on our supplementary replication analyses, we conclude that the cluster structure observed in the NFBC66 reflects a structure in the population, as opposed to an arbitrary grouping of individuals.
